# PAE adsorption on polyelectrolyte-grafted pulp fibers

**DOI:** 10.1515/npprj-2025-0011

**Published:** 2025-10-14

**Authors:** Abdollah Karami, Xiao Wu, Jose Moran-Mirabal, Robert H. Pelton

**Affiliations:** Department of Chemical Engineering, McMaster University, 1280 Main Street West, Hamilton, ON, L8S 4M1, Canada; Department of Chemistry and Chemical Biology, McMaster University, 1280 Main Street West, Hamilton, ON, L8S 4M1, Canada

**Keywords:** adsorption isotherms, PAE, TEMPO, polymer grafting, adsorption analysis

## Abstract

The adsorption of cationic polyamide-amine epichlorohydrin (PAE) paper wet-strength resin onto bleached kraft pulp fibers was increased by grafting hydrolyzed poly(ethylene-alt-maleic anhydride) (PEMAc) onto fiber surfaces before PAE adsorption. The highly negatively charged PEMAc increased the sparse negative charge density on untreated fibers, enhancing PAE adsorption. The adsorption isotherm analysis provided estimates of adsorbed PAE on exterior fiber surfaces, Γ_ex_, as well as the ideality index, *IX*, a new measure of isotherm ideality. *IX* increases when grafting lowers the access of adsorbing polymer to interior surfaces, whereas *IX* decreases when grafting enhances the adsorption capacity of interior surfaces. A library of 18 adsorption isotherms compares PAE and poly(diallyldimethylammonium chloride) adsorption on unmodified pulps, grafted pulps, and TEMPO-oxidized pulps. The grafted pulps include PEMAc polymers modified with pendent short alkyl or poly(ethylene glycol) chains. A grafted PEMAc content of 4.1 mg PEMAc per g of dry fiber tripled the amount of adsorbed PAE on exterior fiber surfaces. About two anionic PEMAc carboxylic acid groups were required for every adsorbed PAE cationic charge. Modification of the PEMAc with pendent chains had little effect. TEMPO oxidation, at the same total fiber charge content, had no positive impact on PAE adsorption.

## 1 Introduction

The adsorption of positively charged, water-soluble polymers onto negatively charged wood pulp fibers is a dominant surface treatment technology in papermaking and related processes. Adding a cationic polymer solution to a stirred aqueous suspension of cellulose fibers yields polymer-coated fibers. The presence of adsorbed polymer can impact paper strength, filler particle retention in the papermaking process, and paper structure (Hubbe et al. 2009; Wågberg 2000; Wågberg and Ödberg 1989). However, the amount of cationic polymer that can adsorb is limited because adsorption tends not to occur beyond a single layer of adsorbed polymer. Efforts to adsorb more cationic polymer in and on fibers include using colloidal polymer electrolyte complexes (Gärdlund et al. 2007), TEMPO-mediated oxidation (Kitaoka et al. 1999; Saito and Isogai 2006; Saito et al. 2006), and layer-by-layer sequential adsorption of oppositely charged polymers (Sandberg and Andreasson 2002; Wågberg et al. 2002). Our recent efforts (Zhang et al. 2021) have focused on grafting poly(ethylene-alt-maleic acid), PEMAc, and some of its derivatives onto exterior fiber surfaces. These polymers have a high negative charge density from the two carboxylic acid groups on every repeat unit. The attachment of PEMAc to fiber surfaces increases the capacity of fibers to adsorb cationic polymers. This contribution focuses on polyamide-epichlorohydrin, PAE, wet-strength resin adsorption onto modified pulp fibers (Ampulski and Neal 1989; Obokata and Isogai 2004). A library of 18 adsorption isotherms is analyzed using new methods, giving insights into the distribution of PAE on and in fiber walls. Before moving to the new experimental results, we summarize our isotherm analysis strategies.

### 1.1 Isotherm analysis

#### 1.1.1 Directly extractable parameters

**Figure 1:**
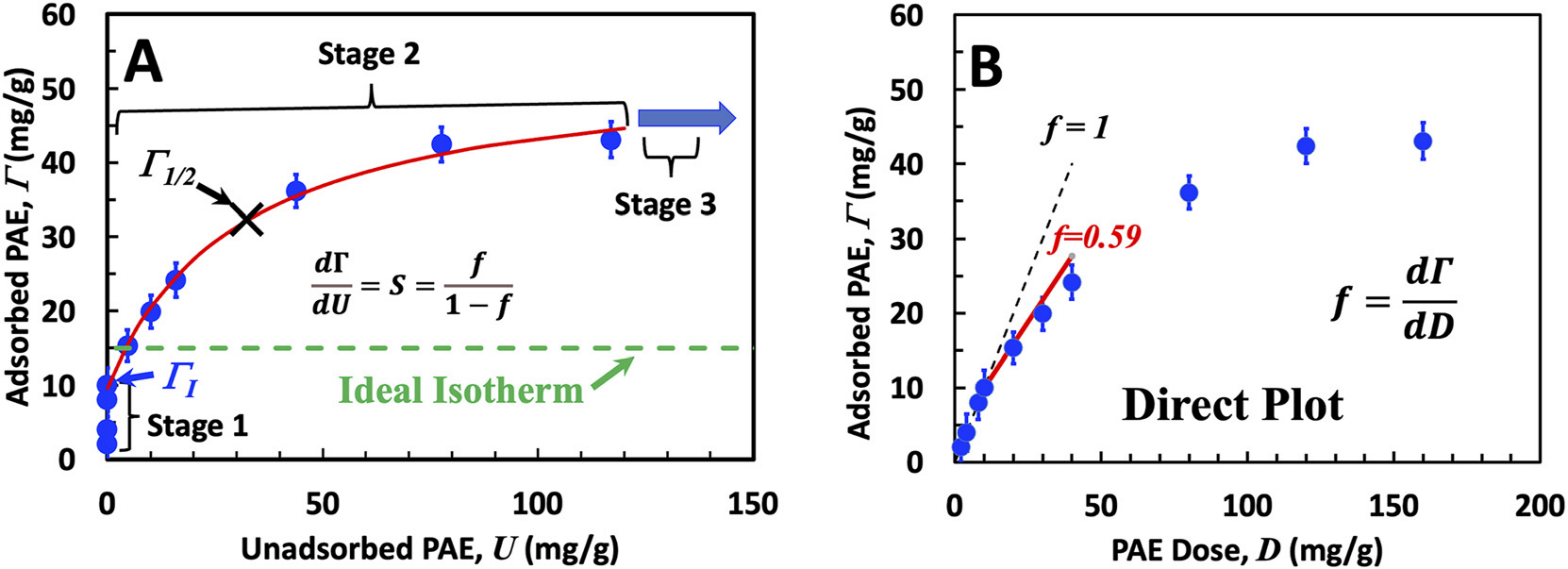
PAE adsorption of fibers with 18 μeq/g L PEMAc (row 8 in Table 1). Axis units, mg of polymer per g of dry fiber. The error bars show the standard deviation (*n*=3).

Starting with an example, Figure 1 shows two views of the same data. Figure 1A is an adsorption isotherm plot of adsorbed polymer Γ as a function of unadsorbed polymer, *U*. A complete list of abbreviations is given after the conclusion section. Figure 1B is the corresponding direct plot of Γ against the dose of adsorbed polymer, *D*. The units of all axes are mg of PAE per g of dry fiber substrate. Both plots are conveniently divided into three stages. Stage 1 is where all the added polymer is adsorbed. The four points on the *y*-axis of the isotherm are in Stage 1. Stage 2 corresponds to some added polymer being adsorbed and some not. Stage 3 is when all accessible adsorption surfaces are saturated. Stage 3 is never reached in most of the isotherms herein.

**Table 1:** Summary of adsorption measurements.

Fiber treatment					Polymer adsorption parameters						
Row	Treatment	^a^EW Da	^b^GP μeq/g	^c^TFC μeq/g	Adsorbing polymer	*f*_max_	*f*_1/2_	^h^Γ_1/2_ μeq/g	Γ_*I*_ μeq/g	Γ_lim_ μeq/g	^g^ *IX*
0^**I**^	None			54.21	^d^PAE-15	0.592	0.152	26.1	7.70	3.15	0.295
1	None			54.21	HDAD	0.120	0.202	2.70	5.22	4.59	0.866
2	TEMPO			69.00	HDAD	0.154	0.116	5.16	4.28	3.62	0.830
3	^f^HPEMAc	94.30	44.0	98.21	HDAD	0.217	0.140	20.6	15.8	12.4	0.768
4^I^	TEMPO			69.00	PAE	0.535	0.163	111	8.51	3.96	0.077
5^I^	None			54.21	^e^PAE	0.541	0.309	56.9	11.1	5.08	0.195
6	HPEMAc	94.30	13.1	67.31	PAE	0.583	0.281	126	17.8	7.45	0.142
7	HPEMAc	94.30	44.0	98.21	PAE	0.706	0.336	187	31.5	9.25	0.168
8	LPEMAc	94.30	17.5	71.71	PAE	0.588	0.224	93.1	27.7	11.4	0.297
9	LPEMAc	94.30	41.0	95.21	PAE	0.767	0.254	117	28.6	6.6	0.243
10	C3DS25	110.20	26.0	80.21	PAE	0.791	0.150	199	26.9	5.6	0.135
11	C3DS50	131.80	25.0	79.21	PAE	0.561	0.165	112	26.1	11.5	0.233
12	C3DS75	161.90	25.0	79.21	PAE	0.587	0.315	92.2	12.2	5.0	0.132
13	PEG3DS25	125.10	23.0	77.21	PAE	0.614	0.345	119	24.7	9.5	0.208
14	PEG3DS50	166.40	20.1	74.31	PAE	0.652	0.285	116	23.0	8.0	0.199
15	PEG3DS75	224.40	17.9	72.11	PAE	0.585	0.253	97.2	17.5	7.3	0.180
16	PEG10DS41	231.00	14.0	68.21	PAE	0.673	0.324	127	15.4	5.0	0.121
17	None			54.21	LDAD	0.376	0.275	13.6	8.27	5.16	0.609

^a^The equivalent weight **(*polymer mass per carboxylic acid*)** of the grafting polymer as sodium salts. ^b^The grafted polymer content. ^c^The total fiber charge from titration. ^d^Low-intensity mixing for a short time. ^e^Standard mixing conditions for rows 1–16, higher-intensity mixing for 30 min. ^f^LPEMAc (60 kDa) HPEMAc (100–500 kDa), all derivatives based on HPEMAc. ^g^*IX* is the ideality index and equals Γ_*I*_/Γ_½_. ^h^Γ(μeq/g) = Γ(mg/g) × 1,000/EW_ad_, where EW_ad_ is the EW of the adsorbing polymer. ^I^Γ_*I*_ and *f*_max_ are based on linear interpolation of data points nearest the *y*-axis.

The red curve in Figure 1A is a least squares fit of the Stage 2 data with the empirical function Eq. (1), a Langmuir equation with a *y*-axis offset of Γ_*I*_. *φ* is the extrapolated maximum adsorption, Γ_max_ (mg/g), and *K* (g/mg) is a fitting parameter. The fitting did not include Stage 1 data on the *y*-axis, and the R^2^ value for the fits reported in the Appendix was greater than 0.98. The fits give three parameters: Γ_*I*_, the apparent *y*-axis intercept, the corresponding slope, *S*_max_, and Γ_½_, the point on the isotherm where Γ = *U* = ½ *D*. The significance of these parameters follows below.

(1) $$\Gamma =\frac{\varphi KU}{1+KU}+\Gamma_{I}$$

The horizontal line in green in Figure 1A is an ideal isotherm obtained when all fractions of the adsorbing polymer have equal access to substrate surfaces. There are few examples of truly ideal isotherms in the fiber technology literature (Pelton et al. 2024). Exclusion of larger molecular weight fractions from interior surfaces and slow adsorption onto these surfaces will result in non-ideal isotherms. The focus of our analysis is exclusion.

The direct plot, Figure 1B, has no new information. However, it is attractive because the slope, *f*, goes from 1 in Stage 1 to 0 in Stage 3. Whereas the isotherm slope, *S*, is infinite in Stage 1 and jumps to a finite value in Stage 2 to become 0 in Stage 3. The material balance, *D* = Γ + *U,* links isotherm and direct plots, which leads to *S* and *f* being related by Eq. (2) (Pelton et al. 2024). Visual inspection of the isotherms in the Appendix suggests that the maximum values of *S* and, thus, *f* occur next to the isotherm *y*-axis. *f*_max_, the maximum direct plot slope in Stage 2 comes from the extrapolated *S*_max_ via Eq. (2). The significance of the isotherm parameters is now summarized.

(2) $$f=\frac{S}{S+1}$$

Γ_*I*_ is the highest adsorption value where all the added polymer is adsorbed. When the adsorbing polymer dose, *D*, is greater than Γ_*I*_, some polymer chains are excluded from interior surfaces. From an applications perspective, Γ_*I*_ is important because it indicates the highest dosage with no wasted polymer.

*f*_max_ is the maximum direct plot slope in Stage 2 and is calculated from the experimental extrapolated isotherm slope, *S*_max_ (Eq. (2)), extrapolated to *U* = 0. The greater the *f*_max_, the more the isotherm is dominated by partitioning smaller chains into fiber interior surfaces. In the case of sequential adsorption, where exterior surfaces are saturated before significant adsorption on interior surfaces, *f*_max_ is an estimate of the maximum mass fraction of the adsorbing polymer adsorbing on interior surfaces of the porous substrate. Experimental *f*_max_ values presented below vary from 0.1 to 0.8.

Other useful parameters extracted from isotherms are *f*_½_ and Γ_½_, which correspond to Γ = *U* = ½ *D*. Γ_½_ is interpolated using the empirical fitting Eq. (1). The direct plot slope *f*_½_ comes from applying Eq. (2) to the isotherm slope at when Γ = *U*. A practical advantage of *f*_½_ and Γ_½_ is the data quality are good. In contrast, there can be challenges in acquiring good data at either extreme of Stage 2 because of analytical chemistry limitations. The fraction *IX* = Γ_*I*_/Γ_½_ is a measure of isotherm ideality we call the isotherm ideality index. With a horizontal ideal isotherm Γ_*I*_/Γ_½_ = 1, we see below that this ratio is substantially less than 1 for polydisperse polymers adsorbing on porous fibers. The lower the *IX*, the less ideal the isotherm.

Γ_max_, the final parameter potentially extracted from isotherm data, is the maximum content of the adsorbed polymer. Indeed, because of the slow diffusion into the interior fiber pores (Horvath et al. 2008; van de Ven 2000). Γ_max_ is often an ill-defined and unreliable experimental quantity.

#### 1.1.2 Estimating maximum adsorbed exterior polymer, Γ_ex_

We define the exterior surfaces as those accessible to all molecular weight fractions of the dosed polymer. The location of exterior surfaces includes the macroscopic fiber surfaces such as those observed with an optical microscope, possibly lumen surfaces, larger accessible pore surfaces, and the surfaces of any fines particles. Therefore, the maximum amount of polymer adsorbed on exterior surfaces, Γ_ex_ is a function of both polymer and pulp fiber properties. If the largest adsorbing polymer molecules are small, our definition of Γ_ex_ includes surfaces inside the fiber wall. Alternatively, if the adsorbing polymers is limited to very long, expanded polymer chains, the exterior surface will be dominated by the outer surface viewed with an optical microscope. Indeed, others have employed high molecular weight adsorbing polymers with low polydispersity to measure Γ_ex_ Because when the polymer adsorption on exterior surfaces is stoichiometric, Γ_ex_ gives a measure of the underlying fiber exterior fiber charge content (Horvath et al. 2006; Wågberg et al. 1987, 1989; Winter et al. 1986). Summarizing, if the largest adsorbing polymer is sufficiently small to enter fiber pores, Γ_ex_ includes polymer on surfaces inside the fiber wall, whereas very large adsorbing polymers will be restricted to macroscopic outer surfaces and the surfaces of any fines particles present. Introduced now are simple approximate expressions for estimating Γ_ex_ values from adsorbing polydisperse polymers.

The topochemical adsorption pathways determine the relationships between Γ_ex_ and the experimental quantities, Γ_*I*_, Γ_max_, and *f*_max_. We propose two limiting topochemical pathways – sequential and simultaneous adsorption. Sequential adsorption saturates the fiber exterior surfaces before significant adsorption occurs on the interior surfaces. Therefore, sequential adsorption implies Γ_ex_ = Γ_*I*_.

With simultaneous adsorption, we assume that adsorption occurs on all accessible surfaces at once, and Γ_ex_ = Γ_me_ where Γ_me_ is calculated by Eq. (3). Note that Γ_me_ < Γ_*I*_ because some of the adsorbed polymer chains at Γ_*I*_ are located in the interior pores. The derivation of Eq. (3), described in a preliminary publication (Pelton et al. 2024), assumes that when the dose equals Γ_*I*_, the adsorbed lower molecular weight polymer can access interior and exterior surfaces simultaneously and is distributed uniformly across the exterior and interior surfaces. Inspection of Eq. (3) reveals that with very high Γ_max_ values, Γ_me_ simplifies to Eq. (4), where Γ_lim_ is the infinite-Γ_max_ limiting case. Γ_lim_ is helpful because it does not require an accurate value for Γ_max_. Next, we will demonstrate where Eq. (4) breaks down for low values of Γ_max_, corresponding to substrates with low interior specific surface areas relative to the exterior surface area – i.e., low porosity substrates.

(3) $${\Gamma_{\text{ex}}=\Gamma }_{\text{me}}=\frac{{\left(1-f_{\max}\right)\cdot \Gamma }_{I}\Gamma_{\max}}{\Gamma_{\max-}f_{\max}\Gamma_{I}}$$

(4) $${\Gamma_{\text{ex}}=\Gamma }_{\mathrm{Lim}}=\left(1-f_{\max}\right)\Gamma_{I}$$

**Figure 2:**
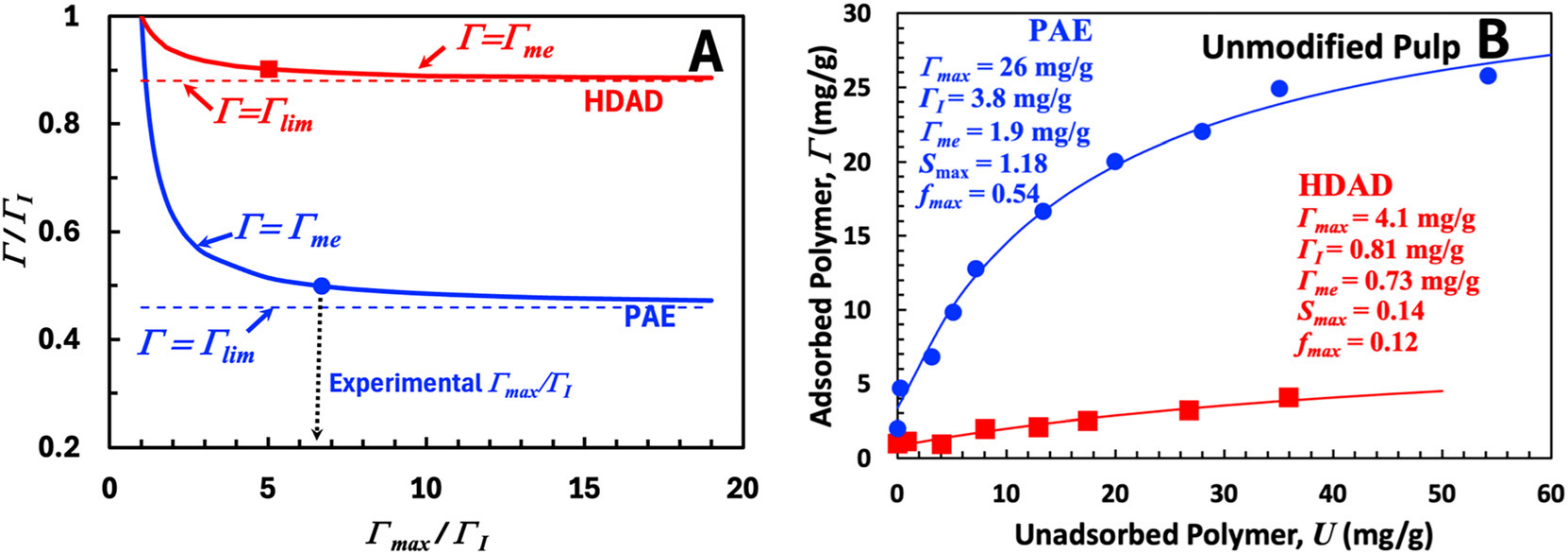
A compares simultaneous adsorption expression Γ_me_ values, Eq. (3), with the corresponding Γ_lim_, Eq. (4), as functions of Γ_max_. Γ_*I*_, and *f*_max_, and the experimental Γ_max_ values come from the isotherms in B (rows 2 and 5 in Table 1).

Figure 2 compares Γ_me_ with the infinite-Γ_max_ limit, Γ_lim_. Plotted are the ratios of Γ_lim_/Γ_*I*_ and Γ_me_/Γ_*I*_ as a function of Γ_max_/Γ_*I*_ for two sets of Γ_*I*_ and *f*_max_ values, taken from the adsorption isotherms for PAE and HDAD on untreated fiber surfaces, shown in Figure 2B. Others have published similar isotherms for PAE (Ampulski and Neal 1989) and HDAD (Wågberg and Hägglund 2001). Shown for each adsorbing polymer in Figure 2A is a curve representing Γ_me_/Γ_*I*_ and a horizontal line representing Γ_lim_/Γ_*I*_. The single symbol on each curve corresponds to Γ_max_ values in the experimental isotherms in B. For PAE and HDAD, Γ_lim_ is a good approximation for ***the simultaneous*** adsorption limit Γ_lim_ ≈ Γ_me_. Summarizing, we propose that Γ_lim_ is the most useful isotherm parameter for estimating the simultaneous adsorption limit because Γ_lim_ is not a function of the difficult-to-measure Γ_max_.

## 2 Materials and methods

### 2.1 Materials

Poly(ethylene-*alt*-maleic anhydride) (PEMA) was purchased from Sigma-Aldrich. The polymer’s MW given by the supplier is 100–500 kDa. Low molecular weight PEMA was supplied by Vertellus, US, as Zama™ E60 (PEMA Mw 60 kDa). Polyamide-amine epichlorohydrin (PAE) resin (Kymene 777 LX, 12.50 % total solid) was provided by Solenis, US.

Three types of poly(diallyldimethyl ammonium chloride), DAD, were employed. Adsorption experiments used HDAD (400–500 kDa) from Sigma and LDAD (8.5 kDa) from Polysciences. Polyelectrolyte titrants (DAD, 0.001 M) and potassium polyvinyl sulfate (PVSK, 0.001 M) were purchased from BTG, Voith, Canada.

The pulp used was Northern Bleached Softwood Kraft (NBSK), provided by CANFOR (British Columbia, Canada). The pulp consisted of a mixture of 40 %–50 % white spruce (*Picea glauca*), 30 %–40 % lodgepole pine (*Pinus contorta*), and 20 %–30 % subalpine fir (*Abies lasiocarpa*).

### 2.2 TEMPO oxidation of pulp fibers

In some experiments, TEMPO-oxidized pulp fibers were used for adsorption. The procedure for preparing TEMPO-mediated oxidation of pulp fibers was adopted from the literature (Saito and Isogai 2006). Saito et al. (2006) First, the pulp fibers (10 g dry) were dispersed in DI water (750 mL) containing 25 mg TEMPO and 250 mg NaBr. Then, 12.5 % NaClO aqueous solution was added to the pulp slurry under continuous mixing, according to the desired mmol NaClO per gram dry pulp. The dose of NaClO was 0.1 mmol NaClO/g dry pulp, which translated to around 50 μL of the NaClO solution. The reaction was carried out at room temperature for 30 min, and the pH of the pulp slurry was maintained above 10.5. Then, the TEMPO-oxidized pulp was filtered and washed thoroughly with water on filter paper and placed in a Buchner funnel. Finally, the wet pulp (10 % consistency) was stored at 4 °C without drying until it was used.

### 2.3 Polymer grafting to pulp fibers

Hydrolyzed poly(ethylene-alt-maleic anhydride), PEMAc, and its derivatives were grafted onto pulp fibers. The details of the PEMAc grafting and product characterization were published (Zhang et al. 2021). The PEMA derivatives were prepared by reaction with n-propylamine or amino-polyethylene glycol. The preparation and solution properties of the derivatives (Wu et al. 2022) and the grafting procedure have been published (Wu et al. 2023). Carboxylic acid contents of the grafted pulps were determined in triplicate by conductometric titration.

### 2.4 Polymer adsorption

Polymer adsorption isotherms were determined by the batch method. DAD or PAE solution was added to **250 mL** of a 0.2 % wt/wt unrefined pulp fiber suspension in a beaker with magnetic stirring. The pH was maintained at 7–8 for 30 min at room temperature, followed by vacuum filtration through a Whatman^®^ qualitative filter paper, Grade 1 (1001-150). The polymer concentration in the filtrate was measured by polyelectrolyte titration using a Mütek PCD-03 detector. When reporting adsorbed amounts as mg/g, the equivalent weights used to convert charge to mass were HDAD (155.3 Da), LDAD (175.6 Da), and PAE (346.6 Da).

Titrations of unadsorbed polymer were performed in triplicate and are reported as a mean and standard deviation (*n*=3). The Supplementary Material file contains a plot of a series of titrations of known PAE concentrations. The titration error was less than 5 % when PAE concentrations were ≥ 5 mg/L, which corresponds to ≥ 2.5 mg of PAE per g of fiber in our adsorption experiments.

The derived isotherm parameters Γ_*I*_, Γ_max_, Γ_½_, *f*_max_*,* and f_½_ were calculated from the isotherm fits to Eq. (1). Few of our isotherms had a clear Stage 3, meaning Γ_max_ values were extrapolated and of questionable accuracy.

We have recently published results of simulations of polydisperse polymer adsorption onto porous substrates (Pelton and Karami 2025). A clear message from the simulations, and inspection of Figure 1B, reveals that *f* in Stage 2 asymptotically approaches *f* = 1 in Stage 1. We define *f*_max_ as the maximum *f* value in Stage 2*.* For all isotherms herein, the maximum occurs when Stage 1 transitions to Stage 2. Where exactly does Stage 2 start? Mathematically, it starts infinitesimally close to Stage 1 and thus equals *f*_max_ = 0.999. *Herein, we report f*_max_ *values* from extrapolating Stage 2 *S* values to the *y*-axis. Therefore, the reported *f*_max_ values are linked to the detection limits of PAE in the unadsorbed polymer solutions. By contrast, the simulations confirm that Γ_*I*_ is not an asymptotic limit and thus is less dependent upon the details of the extrapolation. The values with the least uncertainty are *f*_½_ and Γ_½_ because they correspond to large unadsorbed polymer concentrations that can be accurately measured.

## 3 Results and discussion

Northern softwood bleached pulp fibers were modified to increase the anionic charge densities at cellulose/water interfaces by either TEMPO-mediated oxidation or mainly by grafting high molecular weight carboxylic acid polymers. TEMPO oxidation converts some C6 alcohols on cellulose to a mixture of carboxylic acids and aldehydes (Saito et al. 2006). The chemicals used in TEMPO oxidation are small and thus can access surfaces inside the fiber wall. Consequently, most added carboxylic acid groups are on the dominant interior surfaces (Saito and Isogai 2006). Recent publications show that pulps with significant surface fibrillation and fines content, TEMPO oxidation of outer surfaces dominates (Hillscher et al. 2024; Schäfer et al. 2021).

**Figure 3:**
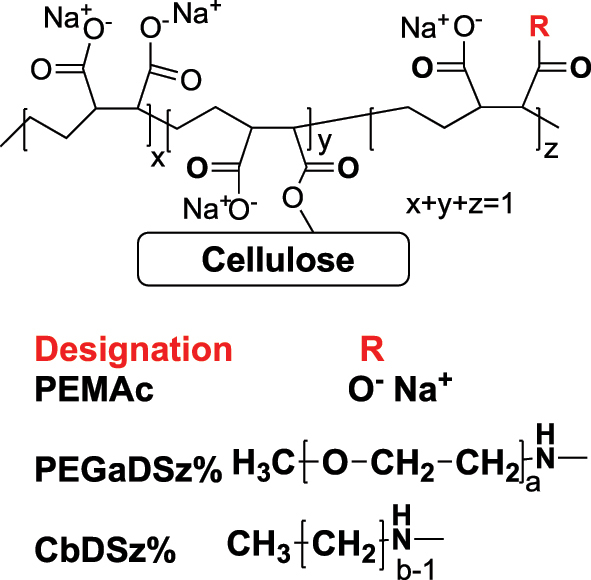
Structures of polymers grafted onto pulp fibers.

All grafting polymers are based on the PEMAc backbone. The details of PEMAc derivative preparation and characterization were previously described in detail (Wu et al. 2023). Figure 3 illustrates the polymer structures. Two series of comb derivatives were prepared – one with “teeth” based on short alkyl chains, called CbDSz%, where b is the number of carbon atoms in the pendant alkyl chains, and z% is the fraction of derivatized repeat units. The other series involves short polyethylene glycol, PEG “teeth,” and is designated PEGaDSz%, where a is the average number of repeat units in the PEG teeth. Every PEMAc repeat unit has two carboxylic acids, whereas every derivatized repeat unit has only one.

In our grafting procedure, dry pulp sheets are wetted with PEMAc or derivatized PEMAc solutions, dried, and cured (Wu et al. 2023). Most of the results involve high MW PEMAc (100–500 kDa), which presumably have limited accessibility inside unbeaten, initially dry fiber walls. Indeed, the isotherm (Figure 1B) for HDAD with a similar MW indicates little adsorption beyond the external surface.

### 3.1 Adsorption database

Eighteen experimental isotherms were measured, which are shown in the Appendix. The isotherm depicted in Figure 1 serves as a typical example. In all cases, the isotherms had non-zero *y*-axis intercepts due to Stage 1 adsorption. In all cases, the maximum isotherm slope in Stage 2, *S*_max_, was near the *y*-axis. Sometimes, *S*_max_ persisted, as seen in the row 4 isotherm, producing a linear isotherm segment near the *y*-axis. However, in most instances, *S* decreased monotonically with increasing dosage throughout Stage 2. Finally, in all cases, the adsorbed polymer contents in the high dosage regimes of Stage 2 greatly exceeded Γ_*I*_, indicating that significantly more polymer was adsorbed on interior surfaces compared to exterior ones.

To simplify the presentation of extensive data reflecting multiple variables, we begin with a summary in Table 1. Each isotherm includes approximately 7–10 PAE or DAD dosages, where each point represents the average of three independent measurements. The left columns in Table 1 summarize the fiber treatments before adsorption, while the right columns summarize the isotherm results. Each row details a specific fiber treatment and the corresponding isotherm parameters for DAD or PAE adsorption. The fiber treatments included none, TEMPO-oxidation, and polymer grafting via PEMAc or its derivatives. The fiber treatment section provides information on the grafting polymer designation, the equivalent weight of the grafting polymer, the grafted polymer GP (μeq/g) content, and the total fiber charge contents TFC (μeq/g), which were measured by conductometric titration.

The right-hand polymer adsorption columns in Table 1 include *f*_max_ and Γ_*I*_, which are extracted from the *y*-axis intercepts, *f*_½_, and Γ_½_ when Γ = *U* = ½ *D*. The two far-right columns give Γ_lim_ (Eq. (4)), the infinite-Γ_max_ simultaneous adsorption estimates for the exterior saturated adsorbed polymer, and *IX*, the ideality index.

Please note that in the isotherms in the appendix, including those in Figure 1, Γ values are expressed as mg of adsorbed polymer per g of dry fiber. However, PAE (equivalent weight 347 Da) and HDAD (equivalent weight 162 Da) have different charge contents. To facilitate comparing results from the two adsorbing polymers, the Γ values in Table 1 are expressed as the corresponding amount of adsorbed cationic charge per g of dry fiber, μeq/g. The two representations of the values are connected by the relationship Γ (μeq/g) = Γ (mg/g) × 1,000/EW_ad_, where EW_ad_ (Da) is the equivalent weight of the adsorbing polymer. We calculated the amount of adsorbed charge by measuring the change in charge content in the supernatant solution.

Row 0 in Table 2 summarizes preliminary experiments that employed a brief 15-min contact time between PAE, a relatively high fiber suspension mass fraction of 0.37 %, and gentle mixing. The adsorption experiments for all other rows in Table 1 were conducted with 30 min polymer fiber contact times and a 0.2 % fiber mass fraction utilizing vigorous magnetic stirring. Thus, comparing rows 0 and 5 illustrates the influence of mixing time and intensity on PAE adsorption on untreated pulp fibers. The *f*_max_ values for rows 0 and 5 were high and closely matched, indicating that *f*_max_ exhibited low sensitivity to mass transport conditions. In contrast, the remaining isotherm parameters depended on mixing time and intensity.

**Table 2:** A compares four estimates of Γ_ex_ based on LDAD, HDAD, and PAE isotherms on untreated fibers.

Adsorbed polymer on exterior surfaces Γ_ex_ (μeq/g)		HDAD, *IX* = 0.87 row 1, μeq/g	LDAD, *IX* = 0.61 row 17, μeq/g	PAE, *IX* = 0.20 row 5, μeq/g
Simultaneous	Γ_ex_ = Γ_me_, Eq. (3)	4.70	6.02	5.53
Simultaneous	Γ_ex_ = Γ_lim_, Eq. (4)	4.59	5.16	5.08
Sequential	Γ_ex_ = Γ_*I*_	5.22	8.26	11.1
Mean of Γ_*I*_ and Γ_lim_		4.90 ± 0.31 (±6 %)	6.71 ± 2.3 (±34 %)	8.08 ± 3.0 (±37 %)

### 3.2 Γ_*I*_, Γ_½_, *f*_max_, *f*_½_, and TFC correlations

**Figure 4:**
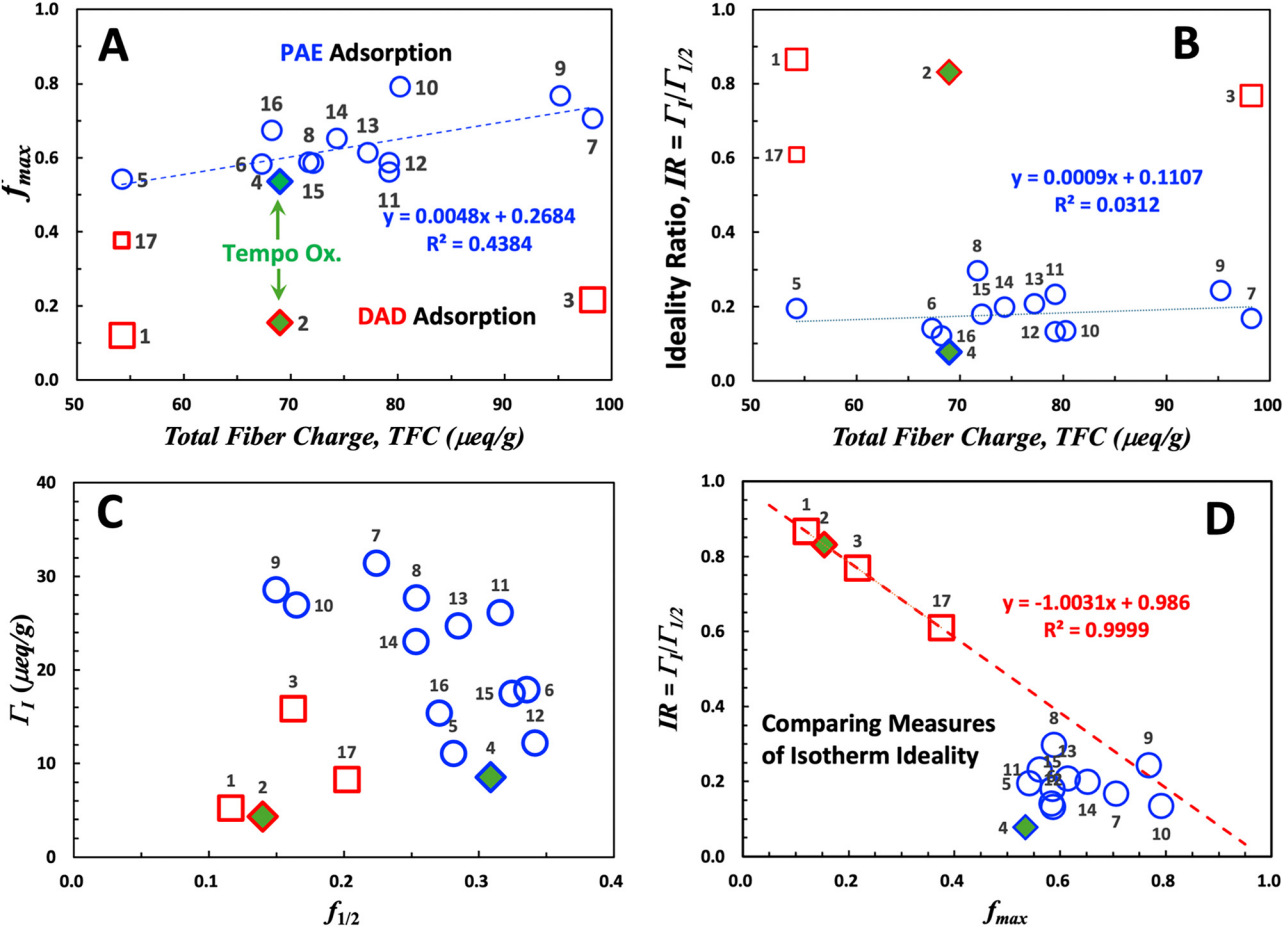
Relationships between the parameters in Table 1. The data point labels correspond to the row numbers in Table 1. The blue points represent PAE adsorption, the red points indicate HDAD isotherms, and the diamonds indicate TEMPO-oxidized pulp.

Before delving into the detailed influences of fiber treatment and graft composition, we explore potential relationships among *f*_max_*, f*_½_*,* Γ_½_, Γ_*I*_, *IX,* and TFC, as tabulated in rows 1–17 of Table 1. Figure 4 shows plots of four combinations of these parameters. In each plot, the square red symbols represent data from DAD isotherms, with the numbers beside the symbols indicating the corresponding row numbers in Table 1. The row 17 point, marked with a smaller symbol, refers to the lower MW LDAD, while the other red symbols correspond to the higher MW HDAD. The blue symbols indicate PAE adsorption isotherms. The diamond symbols represent adsorption onto TEMPO-oxidized fibers. The leftmost points in plots A and B (rows 1, 5, 17) correspond to untreated fibers, while the rightmost points (rows 3, 7, 9) represent fibers grafted with unmodified PEMAc, which introduces the most charges. The fibers grafted with modified PEMAc are located in the middle. Note that every blue PAE datum represents a unique type and quantity of grafted polymer.

The Figure 4A *y*-axis, *f*_max_, is the maximum slope of Stage 2. The red HDAD isotherms (rows 1–3) exhibit the lowest *f*_max_, whereas the PAE *f*_max_ data are significantly higher. Comparing rows 1 and 2, TEMPO oxidation increased the total fiber charge from 54 to 69 μeq/g. However, the *f*_max_ values for the TEMPO pulp results were similar to the corresponding untreated fiber results, specifically rows 1 vs 2 for HDAD adsorption and 4 vs 5 for PAE adsorption.

Figure 4B and D shows ideality indices *(IX)* as functions of total fiber charge and o*f f*_max_*.* Recall that an ideal horizontal isotherm yields *IX* = 1, while polydisperse adsorbing polymers result in lower values. In addition, if we assume sequential PAE adsorption where Γ_ex_ = Γ_*I*_, the IX is also the fraction of adsorbed polymer on the external surfaces when Γ = Γ_½_.

Grafting highly anionic polymers like PEMAc has the potential to increase or decrease *IX*. Grafted high MW PEMAc could block access of low molecular weight adsorbing to interior surfaces, increasing *IX.* Alternatively, lower MW LPEMAc by penetrating more of the fiber wall may increase fiber swelling and the surface charge density on interior surfaces, increasing the adsorption capacity of interior surfaces, decreasing *IX.* The PAE *IX* values in Figure 4B are clustered around ∼0.11, insensitive to underlying fiber charge contents. The absence of dramatic changes in PAE IX due to grafting suggests that PEMAc blocking access to interior pores is insignificant.

Figure 4C shows Γ_*I*_ as a function of *f*_½_*.* There is no apparent relation between the two parameters, suggesting they are sensitive to different physical properties of the system. One goal of grafting is to increase PAE adsorption under conditions resulting in minimal PAE waste. Γ_*I*_ is the highest PAE dose with no unadsorbed (i.e., wasted) polymer. For example, row 4, TEMPO oxidized pulp, and row 6, PEMAc grafted pulp, have nearly equal total charge contents; however, the Γ_*I*_ of the grafted pulp is twice that of TEMPO pulp.

### 3.3 Adsorption on untreated fibers

Table 2 compares four estimates of the exterior adsorbed charge on untreated fibers, Γ_ex_, from the adsorption isotherms of three polymers – LDAD (8 kDa), HDAD (400–500 kDa), and PAE. The *IX* values range from near ideal for HDAD to nonideal for PAE. Focusing first on HDAD, typical of the polymers used to estimate exterior fiber charge, Table 2 reveals that the four HDAD estimates of Γ_ex_ are close, reflecting the low value of *f*_max_ = 0.12. We believe that given the HDAD high MW, Γ_ex_ = Γ_*I*_ = 5.22 μeq/g is the best estimate.

The Γ_ex_ estimates in Table 2 for LDAD and PAE adsorbing onto unmodified fibers were greater than those for HDAD and exhibited more model dependency. When comparing polymers with different molecular weight distributions, it is important to remember that the exterior (i.e., completely accessible) surface area, ESSA (m^2^/g), increases as the highest molecular weight fractions of the adsorbing polymer decrease. According to the supplier, LDAD has an MW of 8 kDa, meaning the largest LDAD chains access a greater area than 500 kDa HDAD chains. Therefore, it is surprising that the Γ_ex_ estimates for the three polymers in Table 2 are similar.

### 3.4 Adsorption on modified fiber surfaces

**Figure 5:**
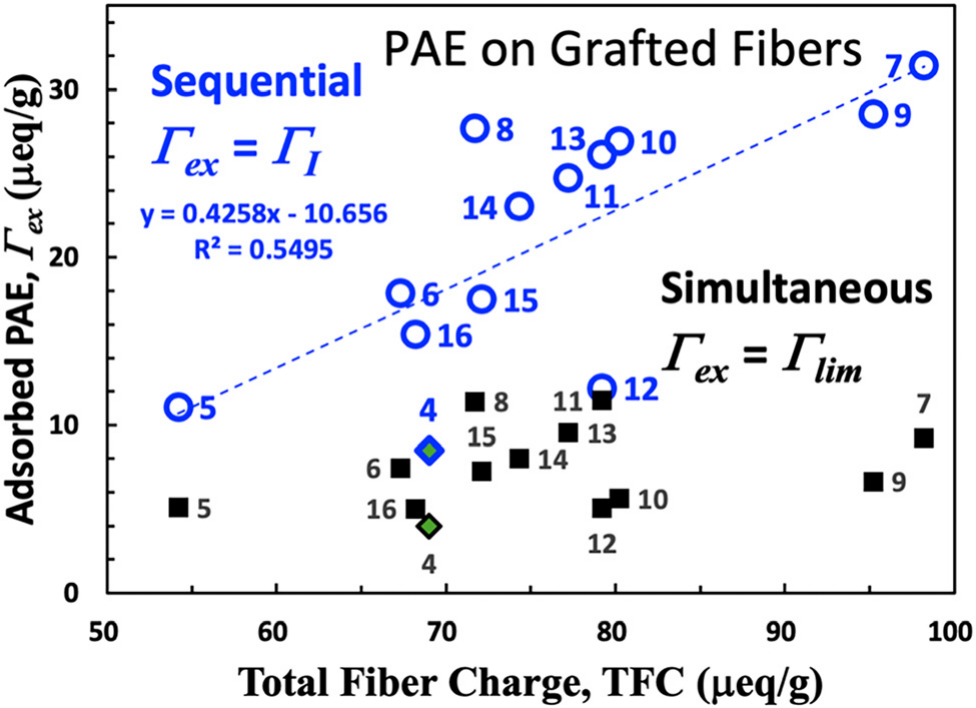
Sequential and simultaneous adsorption limits of Γ_ex_ for PAE adsorption on grafted pulps plus one TEMPO-oxidized pulp. The dashed blue line is the least squares fit, excluding the oxidized pulp.

One incentive for grafting high-molecular-weight PEMAc or its derivatives onto wood pulp fibers is to increase the PAE adsorption capacity on exterior fiber surfaces, i.e., increasing Γ_ex_, ***while minimizing wasted, unadsorbed PAE***. Figure 5 illustrates the two limiting values for Γ_ex_ as a function of the total fiber charge for PAE adsorption on grafted pulps. The simultaneous values are independent of TFC, reflecting that *f*_max_ values are nearly constant. The scatter results from the variability of Γ_*I*_ and *f*_max_ – see Eq. (4). We believe that most grafted HPEMAc and its derivatives are present on the exterior surfaces because HPEMAc has a high molecular weight (100–500 kDa) and is unlikely to penetrate fiber wall pores during impregnation, the first step of grafting. Therefore, grafting should concentrate carboxylic acid groups on the exterior surfaces and thus contribute to Γ_ex_. With this understanding, Γ_ex_ = Γ_*I*_ seems like the most reasonable choice.

If PAE adsorption on grafted fiber surfaces were stoichiometric, we would expect the slope of the blue trendline in Figure 5 to equal 1 instead of 0.43. PAE adsorption on dense grafted PEMAc layers is not stoichiometric. Laine’s study of PAE adsorption on fibers bearing grafted carboxymethyl cellulose (CMC) also revealed that adsorption was not stoichiometric. They reported about 3 μeq/g of CMC per increased μeq/g of PAE (Laine et al. 2002). The impediments to stoichiometric charge balance include the poor registration of relatively sparse cationic groups of PAE with the densely charged PEMAc backbone and hindered access to the interior of large PAE chains to interior carboxylic acid groups in dense grafted PEMAc layers.

Figure 5 includes two types of PEMAc derivatives. Row 10–12 show the influence of hydrophobic n-propyl groups. Rows 10 and 11 are within the primary data cluster, whereas row 12 (C3DS75 grafted) is an outlier with low Γ_ex_ and Γ_*I*_ values. The row 12 polymer had 75 percent of the repeat units in C3DS75 had propyl substituents, making it the most hydrophobic derivative. However, there was no evidence of micellization of the propyl derivative solutions (Wu et al. 2022). At neutral pH, its degree of ionization is about 10 % higher than PEMAc (Wu et al. 2022). It is unclear why row 12 grafted polymer gave low PAE adsorption. We can conclude that there was no evidence for enhanced PAE adsorption due to hydrophobic PEMAc modification.

The PEG3 derivatives, rows 13–16, had polyether chains, and the Γ_ex_-values followed the main trend. The high PEG3 polymer (row 15) and PEG10 polymer (row 16) are slightly below the trend line in Figure 5, whereas the lower PEG3 polymers (rows 13 and 14) are above. PEG modification had little impact on PAE adsorption, emphasizing the importance of electrostatically driven adsorption.

## 4 Conclusions

Our experimental program aimed to understand how grafting the anionic polyelectrolyte PEMAc onto wood pulp fibers affected the adsorption of the cationic wet strength resin PAE. The literature provided little guidance in interpreting nonideal isotherms typical of polydisperse polymers adsorbing onto porous pulp fibers. Therefore, we have developed what we propose as useful strategies for isotherm analysis.

Starting with the pulp fiber substrates, we can identify three types of surfaces on porous adsorption substrates: 1) inaccessible surfaces that cannot be reached by any fraction of the adsorbing polymers; 2) exterior surfaces that are completely accessible to all fractions of the adsorbing polymer, regardless of molecular weight or composition; and 3) interior surfaces that are accessible only to specific fractions of the adsorbing polymer. While these definitions of accessible, interior, and inaccessible surfaces are clear, they reflect the combined properties of both the substrate and the adsorbing polymers.

In papermaking technology and possibly other applications, there is interest in estimating the saturation coverage of adsorbed polymer on exterior surfaces. This contribution advances our earlier analysis (Pelton et al. 2024) by introducing two new elements. The first is Eq. (4), which provides a simplified equation for estimating the amount of adsorbed polymer on exterior surfaces, Γ_ex_, in the limiting case of simultaneous adsorption. Eq. (4) is suitable when the interior accessible surface area is significantly larger than the exterior area, which is typically the case for wood pulp fibers. Three expressions for calculating Γ_ex_ are compared for three polymers: LDAD, HDAD, and PAE, which are adsorbing onto unmodified pulp – see Table 2. For the high MW DAD, commonly used to measure the exterior fiber charge content, the three expressions yield similar values. In this case, setting Γ_ex_ equal to the experimental Γ_*I*_ is a sound choice. However, with the more polydisperse LDAD and PAE, estimates are more sensitive to the chosen model.

The second new element is the isotherm ideality index, *IX* = Γ_*I*_/Γ_½,_ where Γ_½_ represents the amount of adsorbed polymer at the point in the isotherm where Γ = *U* = ½ *D* in Stage 2. An ideal horizontal isotherm has an *IX* of 1, whereas the average *IX* for the PAE isotherms discussed here is 0.11. We propose that when comparing adsorption onto grafted versus ungrafted fibers, the *IX* will increase if grafting limits access to interior surfaces, while *IX* will decrease if grafting enhances the accessibility of interior surfaces through increased swelling or by increasing interior surface charge density.

**Figure 6:**
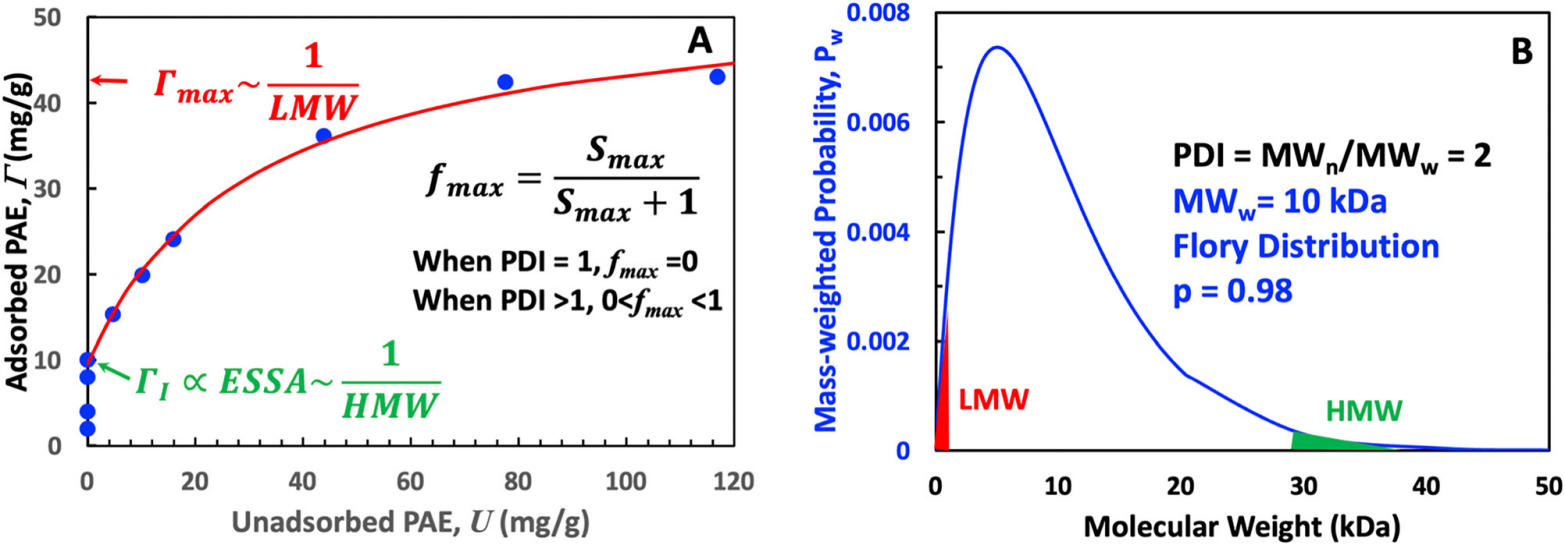
Adsorbing polymer molecular weight distribution properties HMW, LMW, and *Ð* influence isotherm parameters Γ_*I*_, Γ_max_, and *f*_max_. The isotherm data are from Figure 1, and the polymer MW distribution was calculated for an ideal condensation polymer.

Figure 6 illustrates the proposed linkages between the isotherm parameters and the molecular weight distribution of the adsorbing polymer. The adsorption data are real, while the polymer MW distribution was calculated for an ideal condensation polymer. Γ_max_ is dominated by the adsorbing polymer with the lowest molecular weights (LMW) that can access the smallest fiber wall pores. The lower the molecular weight of the LMW fraction, the greater the Γ_max_. The intercept Γ_*I*_ and the external specific surface area of the substrate (ESSA) increase as the molecular weight of the highest MW of the adsorbing polymer decreases. Finally, *f*_max_ is associated with the dispersity (*Ð*) of the adsorbing polymer. If the *Ð* equals 1, indicating a polymer where every chain has the same molecular weight, the isotherm is horizontal, signifying no Stage 2. For typical polymers with a distribution of MWs, the value of *f*_max_ will increase with *Ð* while *IX* decreases.

Grafting PEMAc or its derivatives increased the titratable charge contents, with maximum PEMAc grafting doubling the total fiber charge, TFC. Despite the grafted, high molecular HPEMAc being mainly confined to exterior surfaces where it is most accessible, Figure 5 reveals that about 2 μeq/g of grafted HPEMAc was required to increase the PAE Γ_ex_ by 1 μeq/g. This non-stoichiometric adsorption on grafted polyelectrolytes is consistent with reported PAE adsorption on fibers coated with carboxymethyl cellulose (Laine et al. 2002). Modifying the PEMAc with numerous short pendant PEG or hydrocarbon chains had no significant impact on PAE adsorption compared to unmodified PEMAc. This indicates that electrostatic interactions primarily governed the adsorption of PAE onto our collection of modified fibers. Lastly, the consistency of the isotherm ideality index (Figure 4C) suggests that polymer grafting levels as high as 4 mg/g did not affect PAE accessibility to interior surfaces. From a practical standpoint, grafting 4 mg/g of PEMAc tripled PAE Γ_*I*_, the highest dose that is completely adsorbed.

## Supplementary Material

Supplementary Material

## Abbreviations

### Fiber properties

GP: The grafted polymer content (μeq/g).
SSA: The specific surface area of pulp fibers (m^2^/g).
ESSA: The exterior specific surface area (m^2^/g).
TFC: Total titrated fiber charge, (μeq/g).

### Polymers

C3DSx%: The propylamide pendent PEMAc with x% substitution.
EW: Equivalent weight of adsorbing or grafting polymer (Da).
HDAD: The 400–500 kDa poly(diallyldimethyl ammonium chloride).
LDAD: The 8.5 kDa MW poly(diallyldimethyl ammonium chloride).
LPEMAc: The hydrolyzed 60 kDa MW poly(ethylene-alt-maleic anhydride).
HPEMAc: The hydrolyzed 100–500 kDa poly(ethylene-alt-maleic anhydride).
PAE: The polyamide-amine epichlorohydrin wet strength resin.
*Ð*: The dispersity of the adsorbing polymer, MW_n_/MW_m_.
PEGyDSx%: The PEG amide pendent PEMAc, where y is the average PEG repeat units.

### Adsorption isotherm

#### Measurable dimensionless slopes

f: The slope of the direct adsorption plots.
*f*_max_: The maximum slope of direct adsorption plots in Stage 2.
*f*_1/2_: The slope of direct adsorption plots when Γ/*D* = 0.5.
S: The slopes of isotherm plots.
*S*_max_: The maximum isotherm slope in Stage 2.

#### Measurable isotherm parameters (mg or μeq per g dry fiber)

*D*: The dose of grafting polymer in an adsorption experiment.
*U*: Unadsorbed polymer concentration.
Γ_*I*_: The *y*-axis intercept of the adsorption isotherm.
Γ_½_: Adsorbed polymer when *Γ* = *U* = ½ *D.*
Γ_max_: The maximum measured adsorbed polymer.

#### Curve fitting parameters

*φ*: Γ_Max_ from nonlinear regression (mg/g).
*K*: Fitting constant (g/mg).
R^2^: Measure of regression quality.
RMSE: Is the root mean square error.

#### Derived quantities (mg or μeq per g dry fiber)

*ΙX*: The isotherm ideality index = Γ_*I*_/Γ_½_.
Γ_ex_: The maximum adsorbed polymer on exterior fiber surfaces.
Γ_me_: Γ_ex_ based on the simultaneous adsorption model, Eq. (3).
Γ_lim_: The infinite-Γ_max_ limit of Γ_me,_ Eq. (4).
