# Supplementary material for: PAE adsorption on polyelectrolyte-grafted pulp fibers
Source: Nord Pulp Paper Res J. 2025 Oct 14;41(1):139–50. doi: 10.1515/npprj-2025-0011 (PMC12912036; doi:10.1515/npprj-2025-0011)
Supplement: Supplementary file 1 — Supplementary Material [file j_npprj-2025-0011_suppl_001.pdf]

Supplementary Material - PAE Adsorption on Polyelectrolyte-Grafted Pulp Fibers

Abdollah Karami[1], Xiao Wu[1], Jose Moran-Mirabal [2], and Robert H. Pelton, [1,*]

The figure below illustrates the reproducibility and accuracy of PAE Mütek titrations as a function of PAE concentration. The error in PAE measurement is less than 5% for concentrations ≥ 5 mg/L, corresponding to 2.5 mg/g in our adsorption isotherms. The points are the average of three measurements, and the bar represents ± 1 standard deviation.

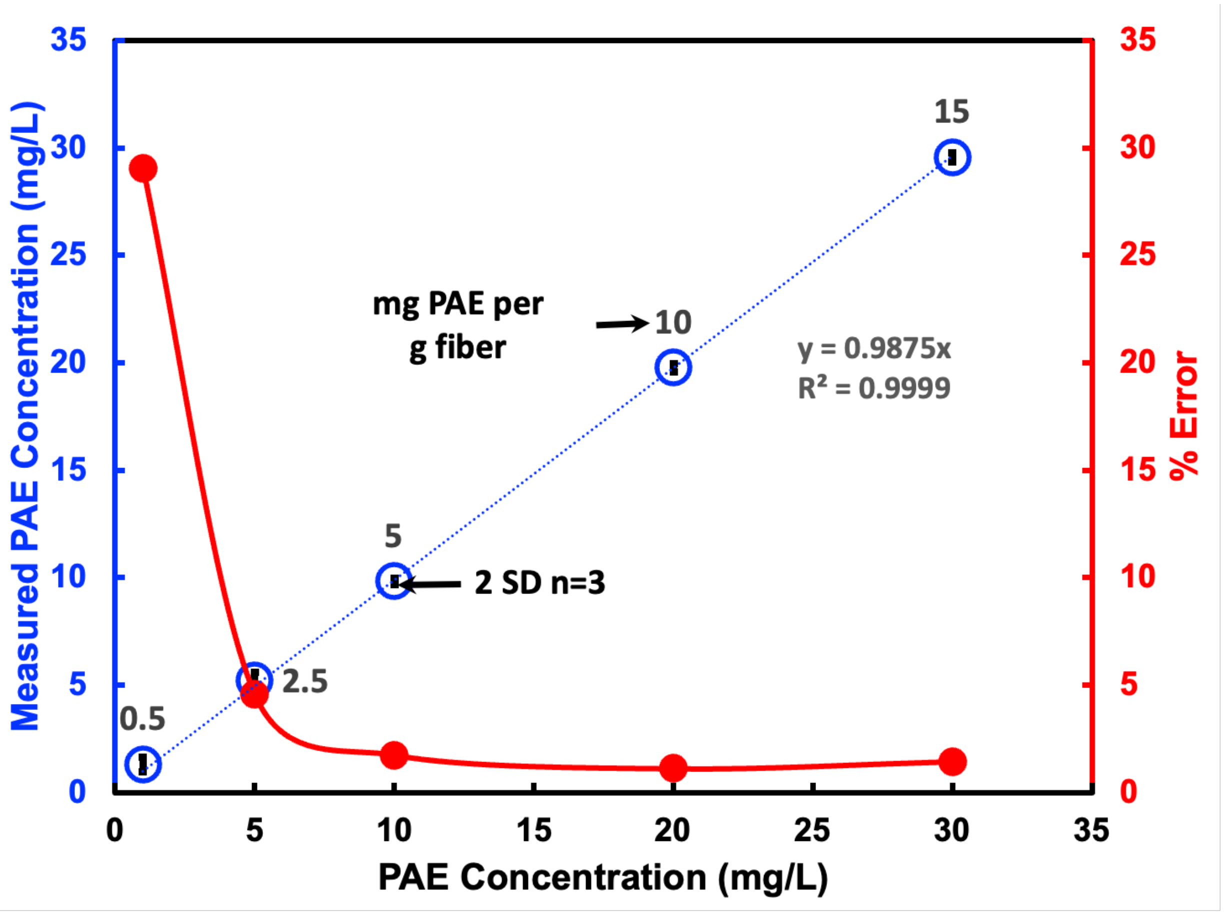

Figure SM1 Mütek charge titration of PAE versus the concentration of PAE.
